# Digital neuropsychological measures by defense automated neurocognitive assessment: reference values and clinical correlates

**DOI:** 10.3389/fneur.2024.1340710

**Published:** 2024-02-15

**Authors:** Huitong Ding, Minzae Kim, Edward Searls, Preeti Sunderaraman, Ileana De Anda-Duran, Spencer Low, Zachary Popp, Phillip H. Hwang, Zexu Li, Kriti Goyal, Lindsay Hathaway, Jose Monteverde, Salman Rahman, Akwaugo Igwe, Vijaya B. Kolachalama, Rhoda Au, Honghuang Lin

**Affiliations:** ^1^Department of Anatomy and Neurobiology, Boston University Chobanian & Avedisian School of Medicine, Boston, MA, United States; ^2^The Framingham Heart Study, Boston University Chobanian & Avedisian School of Medicine, Boston, MA, United States; ^3^Department of Neurology, Boston University Chobanian & Avedisian School of Medicine, Boston, MA, United States; ^4^School of Public Health and Tropical Medicine, Tulane University, New Orleans, LA, United States; ^5^Department of Epidemiology, Boston University School of Public Health, Boston, MA, United States; ^6^Department of Medicine, Boston University Chobanian & Avedisian School of Medicine, Boston, MA, United States; ^7^Department of Computer Science, Faculty of Computing & Data Sciences, Boston University, Boston, MA, United States; ^8^Slone Epidemiology Center, Boston University Chobanian & Avedisian School of Medicine, Boston, MA, United States; ^9^Department of Medicine, University of Massachusetts Chan Medical School, Worcester, MA, United States

**Keywords:** cognitive health, defense automated neurocognitive assessment, digital neuropsychological measures, reference values, clinical correlates

## Abstract

**Introduction:**

Although the growth of digital tools for cognitive health assessment, there’s a lack of known reference values and clinical implications for these digital methods. This study aims to establish reference values for digital neuropsychological measures obtained through the smartphone-based cognitive assessment application, Defense Automated Neurocognitive Assessment (DANA), and to identify clinical risk factors associated with these measures.

**Methods:**

The sample included 932 cognitively intact participants from the Framingham Heart Study, who completed at least one DANA task. Participants were stratified into subgroups based on sex and three age groups. Reference values were established for digital cognitive assessments within each age group, divided by sex, at the 2.5th, 25th, 50th, 75th, and 97.5th percentile thresholds. To validate these values, 57 cognitively intact participants from Boston University Alzheimer’s Disease Research Center were included. Associations between 19 clinical risk factors and these digital neuropsychological measures were examined by a backward elimination strategy.

**Results:**

Age- and sex-specific reference values were generated for three DANA tasks. Participants below 60 had median response times for the Go-No-Go task of 796 ms (men) and 823 ms (women), with age-related increases in both sexes. Validation cohort results mostly aligned with these references. Different tasks showed unique clinical correlations. For instance, response time in the Code Substitution task correlated positively with total cholesterol and diabetes, but negatively with high-density lipoprotein and low-density lipoprotein cholesterol levels, and triglycerides.

**Discussion:**

This study established and validated reference values for digital neuropsychological measures of DANA in cognitively intact white participants, potentially improving their use in future clinical studies and practice.

## 1 Introduction

Alzheimer’s disease (AD) is a debilitating neurodegenerative disorder characterized by progressive cognitive decline. It is a major public health concern, affecting millions of individuals worldwide (1). Unfortunately, to date, there is no definitive cure or highly effective treatment for AD. Given the lack of effective therapeutic options, early detection of the disease is of paramount importance. Timely diagnosis allows for interventions and strategies that can potentially slow down disease progression and improve the quality of life for affected individuals. As such, there is a growing recognition in the scientific and medical communities of the critical need for accurate and early detection methods in the fight against AD.

Traditional neuropsychological (NP) testing has been the primary method for measuring cognitive function, but digital tools have emerged as a promising and convenient alternative (2). Among these tools, the Defense Automated Neurobehavioral Assessment (DANA) stands out as an innovative mobile application developed for the evaluation of neurobehavioral functioning in military personnel (3). DANA was conceptualized to address the pressing need for efficient, objective, and reliable assessment methods capable of monitoring cognitive performance and detecting subtle changes in neurobehavioral functioning over time. Its early deployments within military contexts have yielded valuable insights into the impact of stress-related mental health issues, including conditions such as post-traumatic stress disorder (4), post-concussive symptoms (5), and the effects of blast wave exposure resulting from the use of heavy weapon systems (6). However, DANA’s versatility quickly became apparent, leading to its adoption in a wide range of applications across clinical and research domains. Beyond its original military focus, DANA could be applied in diverse scenarios, including the assessment of traumatic brain injury (7), caregiver burden (8), hypoxic burden at high altitudes (9), and cognition changes in mental health treatments (10). The psychometric properties of the DANA test batteries have been thoroughly assessed and documented, confirming DANA as a reliable and valid tool for neuropsychological assessment (3, 11–14). The adaptability of DANA has transformed it from a specialized military tool into a versatile instrument capable of investigating a wide spectrum of neurocognitive aspects. This expanded utility underscores its significance in addressing various cognitive health-related challenges and outcomes across different clinical and research settings.

One notable challenge is the specialized expertise required to effectively interpret the digital neuropsychological measures obtained through tools like DANA. The outputs of digital tools typically comprise raw numerical values or complex algorithms. Consequently, it necessitates individuals well-versed in neurocognitive functioning and skilled in statistical analysis to extract meaningful insights from these results. This level of expertise is crucial to ensure accurate interpretation and prevent misinterpretation of the findings. Another challenge is the potential absence of established norms or benchmarks for these digital neuropsychological measures. In contrast to traditional neuropsychological tests, which benefit from established norms based on factors such as age and sex, digital neuropsychological measures often lack such reference values. This absence makes the interpretation of results and the establishment of benchmarks for future assessments a complex endeavor. As technology continues to advance and new assessment tools emerge, the development of standardized reference values may require time and concerted effort. Furthermore, the exploration of clinical risk factors linked to these digital neuropsychological measures remains largely unexplored within clinically characterized populations. The absence of established norms and clinical correlates can pose difficulties in determining the clinical implications of the digital measures obtained, emphasizing the importance of addressing this challenge as digital cognitive assessment tools become increasingly prevalent.

Therefore, the primary objective of this study is to establish reference values and investigate the association of clinical risk factors with digital neuropsychological measures derived from DANA in the Framingham Heart Study (FHS). This cohort has undergone a series of DANA tasks and comprehensive characterization of clinical risk factors. We further validated the reference values in participants from the Boston University Alzheimer’s Disease Research Center (BU ADRC).

## 2 Materials and methods

### 2.1 Study population

The FHS is a long-standing community-based prospective cohort study that initiated in 1948. Its primary objective is to identify risk factors of cardiovascular disease as well as dementia within the community. Remarkably, this study has successfully enrolled participants from three distinct generations (15), marking its enduring commitment to advancing our understanding of these critical health issues. In the FHS, cognitive status of participants is determined through a comprehensive dementia review process. This involves a specialized panel, consisting of a neurologist and a neuropsychologist, who meticulously evaluate each case of potential cognitive decline and dementia. Their assessment incorporates a variety of sources, including serial neurologic and neuropsychological evaluations, interviews with caregivers conducted over the phone, medical records, neuroimaging data, and autopsy results when available. The details of dementia review process can be referred to previous studies (16, 17). In this study, 932 cognitively intact white participants from the Framingham Heart Study, including the Offspring, Generation 3, and New Offspring Spouse sub-cohorts, formed the reference cohort.

Additionally, a secondary cohort of 57 cognitively intact white participants who have completed at least one assessment of DANA tasks was recruited from the BU ADRC. These participants were diagnosed based on the National Alzheimer’s Coordinating Center diagnostic procedures (18). Situated in the urban area of Boston, the BU ADRC focuses on older adults living in the community. It is one of 33 ADRCs funded by the National Institute on Aging. These centers contribute data to the National Alzheimer’s Coordinating Center with the aim of fostering collaborative research on AD. More detailed description of the BU ADRC is available in previous publications (19, 20).

### 2.2 Digital neuropsychological measures

This study utilized eight digital measures from three DANA tasks, including the Code Substitution task, the Go-No-Go task, and the Simple Reaction Time task. The Code Substitution task, gauges cognitive flexibility, reflecting how well an individual can transition between tasks or ideas. The Go-No-Go task evaluates inhibitory control, i.e., the ability to inhibit automatic or overlearned responses. The Simple Reaction Time task is a straightforward assessment of reaction speed, focusing on the response time to a given stimulus. For more information on these digital cognitive assessment methods, please refer to the study details (3). Participants will be asked to complete these tasks on a tablet provided to them by study staff during the in-clinic study visit. The details of study design and participation rate can be found in prior study (2). All touch screen and stylus responses will be recorded through the Linus Health app installed on the tablet. The tasks are composed of several trials during each session. Distinct files are produced for each task, encapsulating the various trials that make up one complete round of the cognitive assessment. The FHS and BU ADRC operate independent infrastructure pipelines while utilizing a shared database server, where data is stored in separate databases. For each task, through structured query language, data from individual trials is converted into aggregated examination datasets, with each dataset representing the mean value of the trials. This transformation includes recalculating metrics, conducting quality control, deriving extra metrics, and ensuring uniformity between the two cohorts. For each participant, the first examination of each DANA test was included in this study.

### 2.3 Statistical analyses

We derived reference values in the FHS cohort and stratified participants by sex and age groups: younger than 60 years old (<60), between 60 and 69 years (60–69), and aged 70 years or older (≥70). We employed t-tests to examine sex differences in digital neuropsychological measures. Additionally, one-way analysis of variance (ANOVA) was used to investigate the variations in these measures across different age groups. The reference values for each digital neuropsychological measure were defined as the median (2.5th and 97.5th percentiles) in each stratified group (21, 22). We also examined the association between digital cognitive measures and clinical factors by a backward elimination approach. These clinical factors included age, sex, body mass index, Mini-Mental State Examination (MMSE), smoking, hypertension treatment, systolic blood pressure (SBP), diastolic blood pressure (DBP), total cholesterol (TC), low-density lipoprotein cholesterol (LDL), high-density lipoprotein cholesterol (HDL), the TC to HDL ratio, blood glucose levels, triglyceride levels, diabetes treatment, as well as prevalent cardiovascular disease, myocardial infarction (MI), congestive heart failure (CHF), and stroke. Our analytical process initiated with the inclusion of all these clinical factors in the linear regression model. We then assessed the association of each factor with digital measures. Subsequently, we identified and removed the factor with the least significant association, followed by a reassessment of the relationships between the remaining clinical factors and digital measures. This stepwise elimination procedure was repeated until all remaining clinical factors achieved at least a nominal level of significance (*p* < 0.05). In all our models, we forced age and sex as covariates to account for their potential confounding effects on the observed associations.

## 3 Results

### 3.1 Cohort descriptive

In this study, we included 932 participants from the FHS as the reference cohort (mean age 63.0 ± 9.4 years, range 39.0–88.0 years, 57.3% women). Their clinical characteristics detailed in Table 1. Of these participants, 66.6% had received college education or higher. In addition, we included 57 individuals from BU ADRC as the validation cohort (mean age 74.5 ± 8.7 years, range 52.1–92.5 years, 64.9% women). Among them, 80.7% had a college education or higher.

**Table 1:** Demographics of the reference samples in FHS and BU ADRC cohorts.

Variable	FHS (*n* = 932)	BU ADRC (*n* = 57)
Age (years), mean (SD)	63.0 ± 9.4	74.5 ± 8.7
Women, *n* (%)	534(57.3)	37(64.9)
**Education, *n* (%)**		
No high school	4(0.4)	0
High school	82(8.8)	2(3.5)
Some college	225(24.1)	9(15.8)
College and higher	621(66.6)	46(80.7)

Values are given as *n* (%) for dichotomous variables and mean ± SD for continuous variables.

### 3.2 Reference values of digital neuropsychological measures

**Figure 1:**
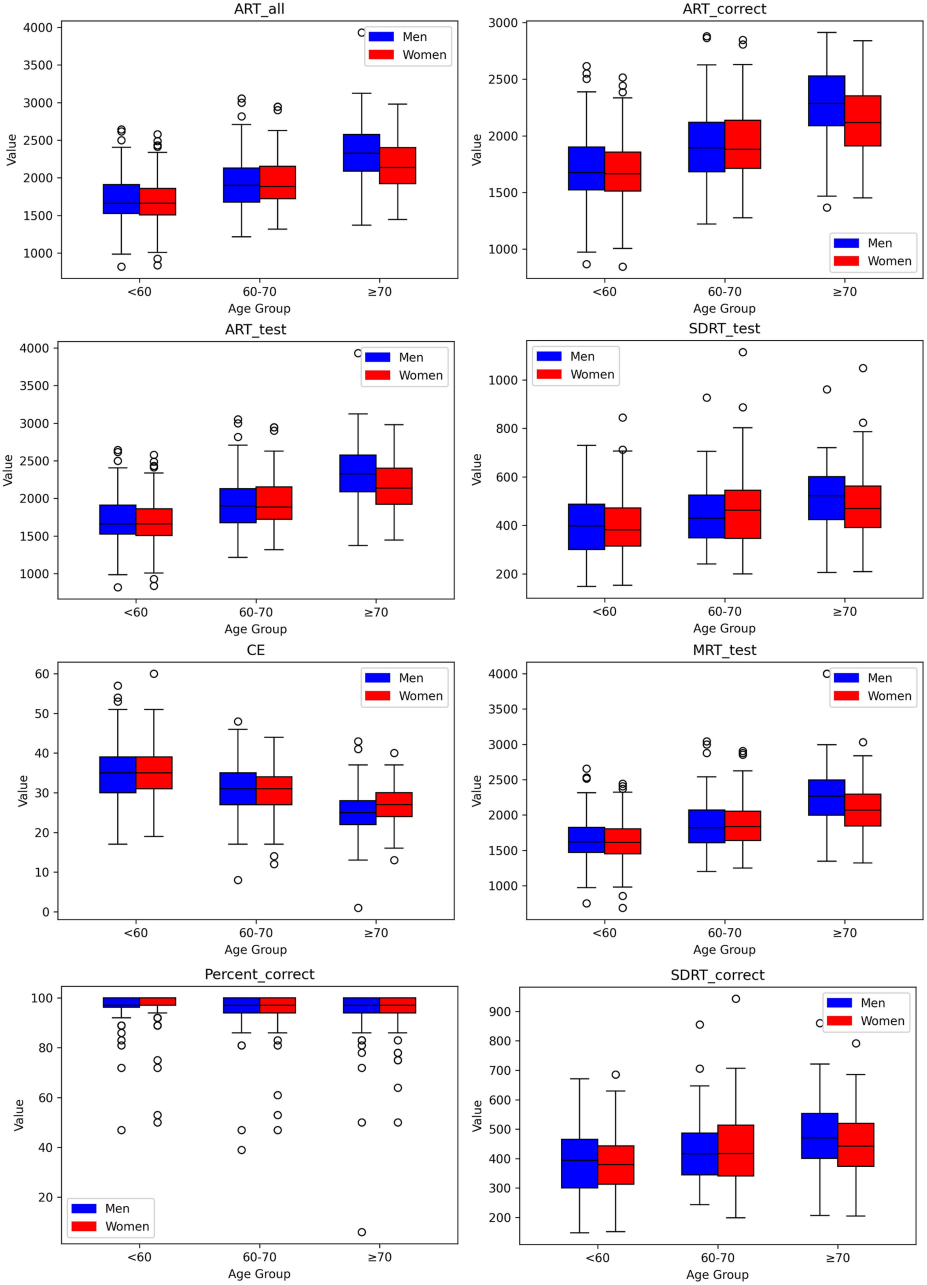
Box-and-whisker plots showing distributions of digital measures of Code Substitution task by age group for men and women. Each box encompasses the middle 50% of the dataset, specifically the second and third quartiles. The reference value (median) is indicated by the internal horizontal line. Whiskers extend from the boxes to the highest and lowest values within 1.5 times the IQR from the upper and lower quartiles, respectively.

**Figure 2:**
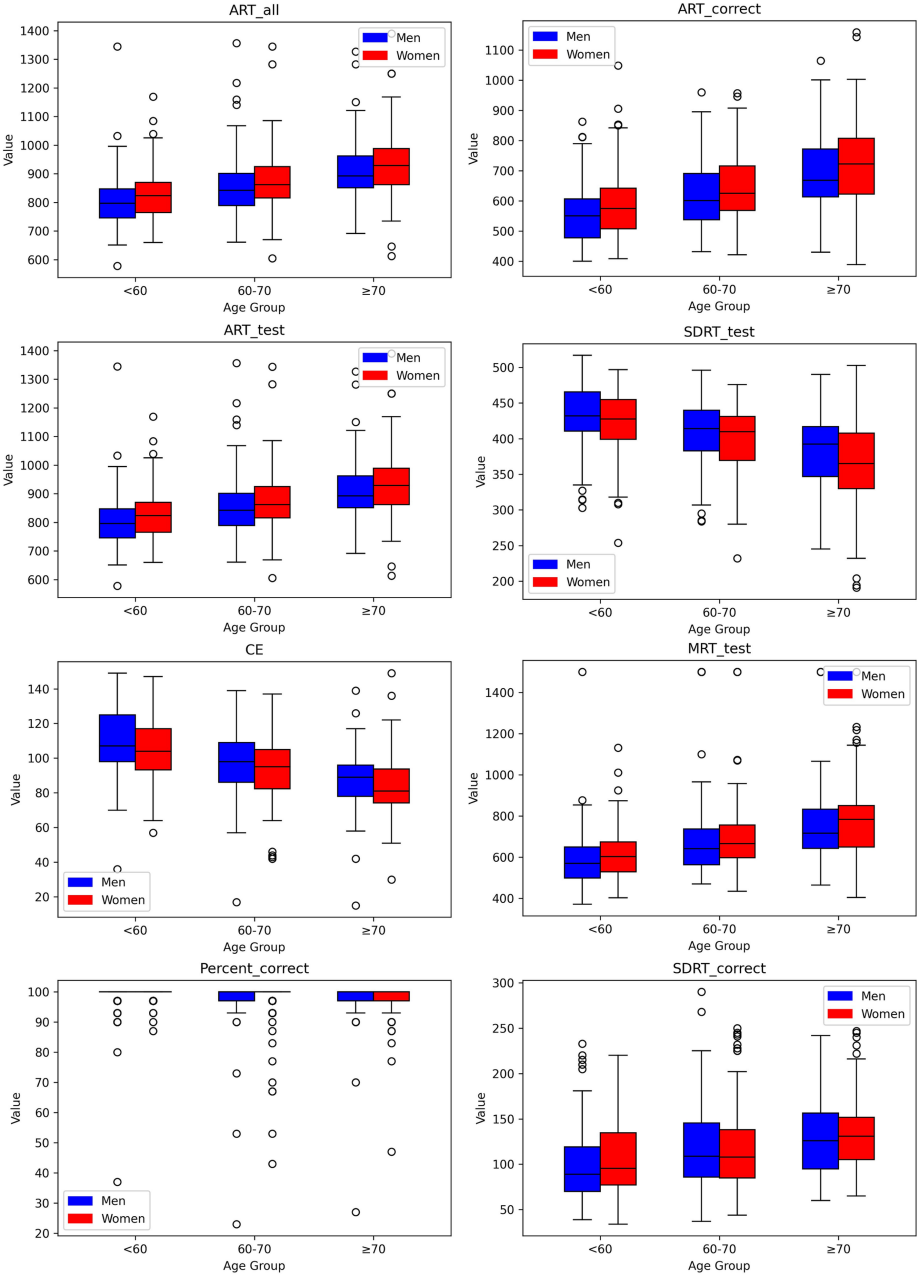
Box-and-whisker plots showing distributions of digital measures of Go-No-Go by age group for men and women. Each box encompasses the middle 50% of the dataset, specifically the second and third quartiles. The reference value (median) is indicated by the internal horizontal line. Whiskers extend from the boxes to the highest and lowest values within 1.5 times the IQR from the upper and lower quartiles, respectively.

**Figure 3:**
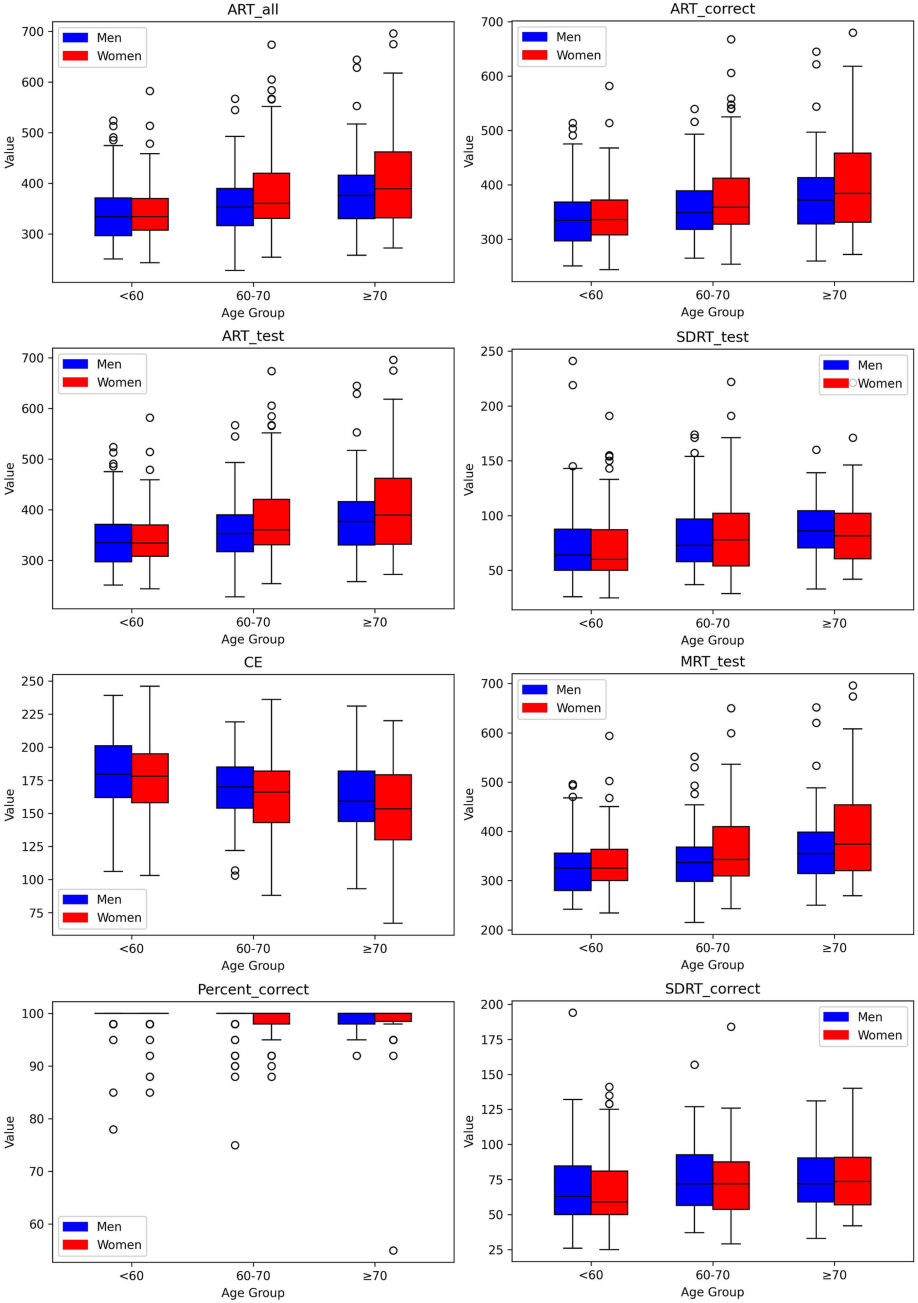
Box-and-whisker plots showing distributions of digital measures of Simple Reaction Time by age group for men and women. Each box encompasses the middle 50% of the dataset, specifically the second and third quartiles. The reference value (median) is indicated by the internal horizontal line. Whiskers extend from the boxes to the highest and lowest values within 1.5 times the IQR from the upper and lower quartiles, respectively.

**Figure 4:**
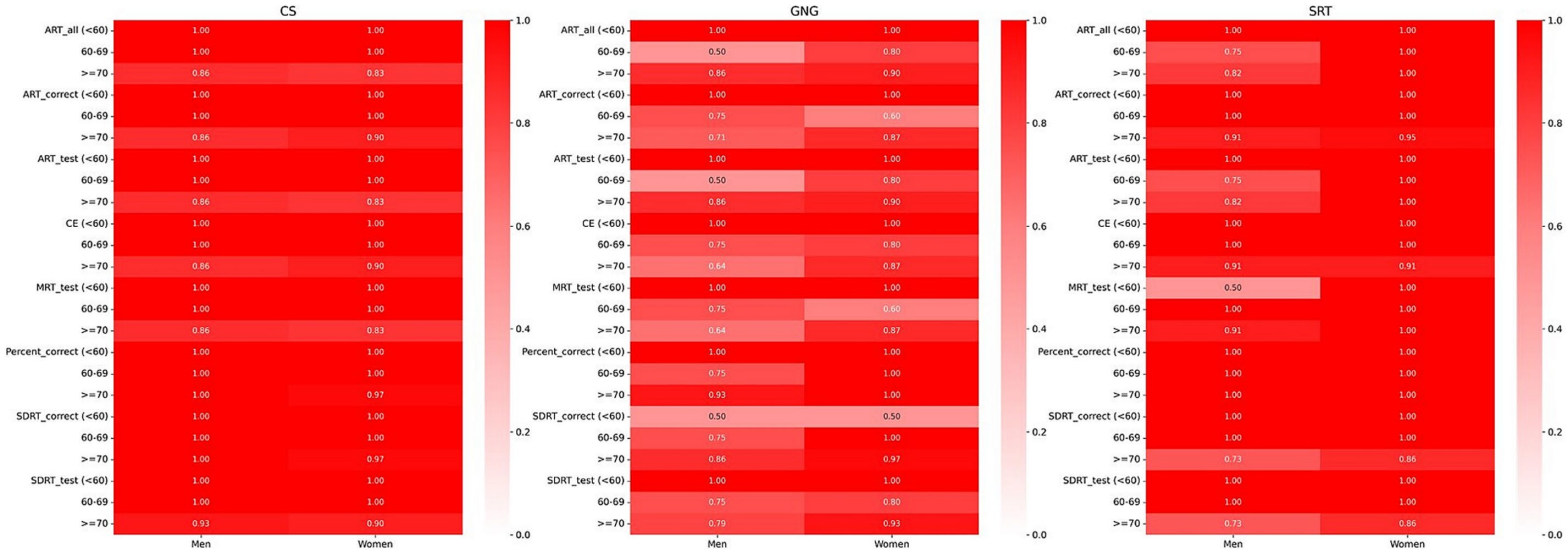
Heatmap illustrating the percentage of BU ADRC participants who scored within the normal range on digital measures, segmented by sex and age and confined to the 2.5th to 97.5th percentile range. A deep red color signifies that 100% (a proportion of 1) of the participants in that particular demographic and digital measures are within the normal range, set between the 2.5th and 97.5th percentiles. Conversely, a white color denotes a 0% proportion, meaning that none of the participants in the group fell within the normal range for that specific digital measure. Shades of lighter red indicate proportions between 0 and 1, reflecting the varying percentages of participants within the normal range.

We established reference values for digital neuropsychological measures across three DANA tasks. These values, including the upper (97.5th quantile), median (50th quantile), and lower (2.5th quantile) reference limits, have been categorized by three distinct age intervals for both men and women, as presented in Tables 2–4. Among participants aged under 60, we observed that in the Code Substitution task, the median (2.5th percentile, 97.5th percentile) average response time for all correct test trials (ART_correct) was 1,674 ms (1,147, 2,377) for men and 1,664 ms (1,188, 2,321) for women. Interestingly, in the Code Substitution task, women exhibited shorter reaction times, indicative of better cognitive function when compared to men within the same age group [*t*(930) = 2.14, *p* = 0.03]. However, men displayed quicker reaction times than women in the Go-No-Go [*t*(904) = 2.28, *p* = 0.02] and Simple Reaction Time tasks [*t*(450) = 1.99, *p* = 0.05]. Furthermore, our findings revealed that reaction times tend to slow down with increasing age (ANOVA, *F* = 178.77, *p* = 2.1 × 10^−66^), a phenomenon observed in both males (ANOVA, *F* = 91.75, *p* = 1.9 × 10^−33^) and females (ANOVA, *F* = 89.34, *p* = 3.6 × 10^−34^). These trends and age-related variations are illustrated in Figures 1–3, offering a comprehensive overview of the distribution of digital neuropsychological measures across different age groups in men and women. For a comprehensive summary of the median digital measures across the three DANA tasks, please refer to Supplementary Tables S1–S3. Furthermore, we categorized the participants into two education groups: those without a college degree and those with college or higher education. Significant differences were found in digital measures between these groups (*t*-test, all *p* < 0.05). The reference values of these two groups can be found in Supplementary Tables S4–S9. Skewness estimates corresponding to partitions in Tables 2–4 are provided in the Supplementary Table S10. Participants with college and higher degree have faster reaction time than the participants with no college degree. Figure 4 represents the proportion of participants from the BU ADRC validation cohort who fall within the normal range (between the 2.5th and 97.5th percentiles) of each digital cognitive measure. The heatmap is stratified by sex and age groups. While the reference values for some subgroups’ digital measures do not fully encompass the validation cohort, most of the measures are within the range of reference values.

**Table 2:** Reference values of digital measures in Code Substitution task.

Digital measure	Age group	Men					Women				
		2.5%	25%	50%	75%	97.5%	2.5%	25%	50%	75%	97.5%
ART_all	<60 (*n* = 338)	1,147	1,525	1,660	1,912	2,386	1,193	1,508	1,661	1,859	2,335
	60–69 (*n* = 378)	1,370	1,677	1,898	2,128	2,692	1,402	1,720	1,884	2,151	2,584
	≥70 (*n* = 216)	1,600	2,087	2,325	2,577	3,034	1,637	1,920	2,137	2,402	2,792
ART_correct	<60	1,147	1,521	1,674	1,901	2,377	1,188	1,512	1,664	1,855	2,321
	60–69	1,376	1,684	1,893	2,120	2,571	1,402	1,714	1,880	2,136	2,490
	≥70	1,607	2,090	2,285	2,528	2,854	1,641	1,912	2,116	2,354	2,754
ART_test	<60	1,147	1,525	1,660	1,912	2,386	1,193	1,508	1,662	1,860	2,335
	60–69	1,370	1,677	1,898	2,128	2,691	1,402	1,720	1,884	2,150	2,584
	≥70	1,600	2,086	2,325	2,577	3,034	1,637	1,920	2,137	2,402	2,792
MRT_test	<60	1,116	1,470	1,618	1,824	2,311	1,134	1,450	1,610	1,806	2,250
	60–69	1,274	1,608	1,819	2,070	2,536	1,336	1,638	1,836	2,054	2,533
	≥70	1,568	1,998	2,269	2,498	2,968	1,608	1,843	2,071	2,294	2,752
SDRT_test	<60	187	300	398	487	673	227	314	382	472	673
	60–69	249	348	429	525	680	240	346	463	545	706
	≥70	277	424	522	601	709	241	391	470	562	782
CE	<60	24	30	35	39	51	25	31	35	39	49
	60–69	20	27	31	35	42	21	27	31	34	43
	≥70	17	22	25	28	36	17	24	27	30	36
Percent_correct	<60	84	96	97	100	100	89	97	100	100	100
	60–69	86	94	97	100	100	86	94	97	100	100
	≥70	75	94	97	100	100	75	94	97	100	100
SDRT_correct	<60	188	301	394	466	624	227	313	380	443	582
	60–69	250	345	416	486	633	233	341	417	514	650
	≥70	273	401	470	553	634	241	374	442	519	646

ART_all, average response time for all trials (ms); ART_correct, average response time for all correct test trials (ms); SDRT_correct, standard deviation of response time for correct test trials (ms); ART_test, average response time for all test trials (ms); MRT_test, median response time for all test trials (ms); SDRT_test, standard deviation of response time for all test trials (ms); CE, cognitive efficiency value (measure of both speed and accuracy); Percent_correct, percent of trials with correct responses within allocated time.

**Table 3:** Reference values of digital measures in Go-No-Go task.

Digital measure	Age group	Men					Women				
		2.5%	25%	50%	75%	97.5%	2.5%	25%	50%	75%	97.5%
ART_all	<60 (*n* = 333)	675	745	796	847	989	701	765	823	870	1,002
	60–69 (*n* = 365)	717	789	842	901	1,072	725	815	862	925	1,044
	≥70 (*n* = 208)	736	851	892	962	1,141	738	861	928	988	1,183
ART_correct	<60	406	478	551	606	789	436	508	575	642	806
	60–69	462	538	601	691	837	473	568	625	716	862
	≥70	507	613	668	772	993	509	622	722	808	994
ART_test	<60	675	745	796	847	989	701	765	823	870	1,002
	60–69	717	789	842	902	1,072	724	815	862	925	1,044
	≥70	736	851	892	962	1,141	738	862	928	988	1,183
MRT_test	<60	425	498	569	650	840	454	528	603	674	852
	60–69	482	562	641	736	972	487	596	666	756	943
	≥70	532	642	716	833	1,058	535	649	784	851	1,178
SDRT_test	<60	330	410	432	466	498	337	399	428	455	482
	60–69	310	383	414	440	479	305	369	410	431	458
	≥70	264	347	392	417	463	227	330	365	408	466
CE	<60	74	98	107	125	142	73	93	104	117	136
	60–69	67	86	98	109	125	67	82	95	105	122
	≥70	59	78	89	96	116	54	74	81	94	118
Percent_correct	<60	90	100	100	100	100	97	100	100	100	100
	60–69	90	97	100	100	100	78	100	100	100	100
	≥70	90	97	100	100	100	86	97	100	100	100
SDRT_correct	<60	46	70	89	119	208	54	77	96	135	204
	60–69	62	86	109	146	215	55	85	108	138	228
	≥70	69	95	126	156	215	69	105	131	152	233

ART_all, average response time for all trials (ms); ART_correct, average response time for all correct test trials (ms); SDRT_correct, standard deviation of response time for correct test trials (ms); ART_test, average response time for all test trials (ms); MRT_test, median response time for all test trials (ms); SDRT_test, standard deviation of response time for all test trials (ms); CE, cognitive efficiency value (measure of both speed and accuracy); Percent_correct, percent of trials with correct responses within allocated time.

**Table 4:** Reference values of digital measures in Simple Reaction Time task.

Digital measure	Age group	Men					Women				
		2.5%	25%	50%	75%	97.5%	2.5%	25%	50%	75%	97.5%
ART_all	<60 (*n* = 149)	257	297	335	371	498	267	308	334	370	479
	60–69 (*n* = 198)	277	317	353	390	492	262	331	361	420	573
	≥70 (*n* = 105)	260	330	377	416	602	291	332	389	462	662
ART_correct	<60	258	297	334	368	495	267	308	336	372	468
	60–69	277	318	348	389	487	263	328	359	412	552
	≥70	266	328	372	414	595	298	331	384	458	617
ART_test	<60	258	297	334	371	498	267	308	334	370	479
	60–69	277	317	353	390	492	262	331	360	420	573
	≥70	260	330	377	416	602	291	332	390	462	662
MRT_test	<60	249	280	325	356	477	255	300	325	363	468
	60–69	263	298	336	368	489	249	310	343	409	536
	≥70	251	314	355	398	590	271	320	374	454	659
SDRT_test	<60	30	50	64	88	169	30	50	60	87	154
	60–69	39	58	73	97	156	35	54	78	102	161
	≥70	37	70	86	104	138	44	60	82	102	165
CE	<60	115	162	180	201	233	125	158	178	195	224
	60–69	122	154	170	185	213	102	143	166	182	226
	≥70	99	144	159	182	218	85	130	154	179	200
Percent_correct	<60	92	100	100	100	100	92	100	100	100	100
	60–69	90	100	100	100	100	92	98	100	100	100
	≥70	95	98	100	100	100	93	98	100	100	100
SDRT_correct	<60	30	50	63	85	132	30	50	59	81	129
	60–69	39	56	72	93	125	35	54	72	88	122
	≥70	36	59	72	90	123	44	57	74	91	131

ART_all, average response time for all trials (ms); ART_correct, average response time for all correct test trials (ms); SDRT_correct, standard deviation of response time for correct test trials (ms); ART_test, average response time for all test trials (ms); MRT_test, median response time for all test trials (ms); SDRT_test, standard deviation of response time for all test trials (ms); CE, cognitive efficiency value (measure of both speed and accuracy); Percent_correct, percent of trials with correct responses within allocated time.

### 3.3 Association of digital neuropsychological measures with clinical risk factors

The same digital cognitive measures observed across all three DANA tasks demonstrate distinct clinical correlates. Notably, for the average response time, it exhibited distinct associations within each task. In the Code Substitution task, the average response time for all correct test trials (ART_correct) was positively associated with diabetes and total cholesterol, and negatively associated with LDL, HDL, and triglycerides. Conversely, in Go-No-Go task, ART_correct was positively associated with blood glucose, and negatively associated with SBP and MMSE. Lastly, in the Simple Reaction Time task, it exhibited a positive association with DBP and the prevalence of MI and stroke, while negatively associated with the prevalence of CHF. In the Code Substitution task, three digital neuropsychological measures demonstrated significant associations with four or more clinical factors. The Go-No-Go task revealed that two digital measures were significantly linked to three clinical risk factors. Meanwhile, the Simple Reaction Time task had seven digital measures significantly associated with three or more clinical risk factors. For a comprehensive breakdown of the clinical factors significantly related to each digital neuropsychological measure within the three DANA tasks, refer to Tables 5–7. The model’s Akaike information criterion (AIC) value for each iteration is depicted in Supplementary Figures S1–S3.

**Table 5:** Multiple regression coefficients for digital measures of Code Substitution task.

Clinical factors	ART_all	ART_correct	ART_test	MRT_test	SDRT_test	Percent_correct	SDRT_correct
Diabetes	230.3 (20.7, 440.0) 0.03	225.6 (34.2, 417.1) 0.02	230.3 (20.7, 439.9) 0.03	231.0 (18.7, 443.2) 0.03			
Total cholesterol		182.5 (9.6, 355.4) 0.04			101.4 (29.3, 173.5) 0.01		62.0 (3.3, 120.7) 0.04
HDL		−182.3 (−354.7, −9.8) 0.04			−101.7 (−173.7, −29.8) 0.01		−62.5 (−121.1, −3.9) 0.04
Triglycerides		−36.9 (−71.5, −2.3) 0.04			−20.4 (−34.9, −6.0) 0.01		−12.8 (−24.5, −1.0) 0.03
LDL		−182.1 (−354.9, −9.3) 0.04			−101.8 (−173.9, −29.8) 0.01		−62.2 (−120.8, −3.5) 0.04
SBP						0.1 (0.0, 0.3) 0.03	

The values are presented as coefficient, 95% confidence interval, *p*-value. Blank cells indicate that the corresponding clinical factors were not statistically significant and were therefore removed from the final backward models. Regression coefficients are presented per unit for continuous variables or as the presence of a condition for binary variables. Age and sex were forced as covariates in all the models. HDL, high-density lipoprotein cholesterol; LDL, low-density lipoprotein cholesterol; SBP, systolic blood pressure.

**Table 6:** Multiple regression coefficients for digital measures of Go-No-Go task.

Clinical risk factors	ART_all	ART_correct	ART_test	MRT_test	SDRT_test	CE	Percent_correct
SBP		−1.8 (−3.4, −0.2) 0.03					
MMSE	−26.7 (−47.8, −5.7) 0.01	−28.6 (−52.4, −4.7) 0.02	−26.7 (−47.7, −5.6) 0.01	−49.2 (−79.7, −18.6) 0.00	11.9 (1.8, 22.0) 0.02	4.9 (1.7, 8.0) 0.00	3.1 (1.5, 4.7) 0.00
Diabetes	132.8 (64.7, 200.8) 0.00		132.8 (64.7, 200.8) 0.00	226.7 (127.9, 325.5) 0.00		−17.4 (−27.7, −7.1) 0.00	−6.4 (−11.6, −1.3) 0.02
Blood glucose		2.4 (1.1, 3.6) 0.00			−0.9 (−1.4, −0.4) 0.00		
Ratio_TC-HDL_							−1.7 (−3.2, −0.2) 0.03

The values are presented as regression coefficient, 95% confidence interval, *p*-value. Blank cells indicate that the corresponding clinical factors were not statistically significant and were therefore removed from the final backward models. Regression coefficients are presented per unit for continuous variables or as the presence of a condition for binary variables. Age and sex were forced as covariates in all the models. SBP, systolic blood pressure; MMSE: Mini-Mental State Examination; Ratio_TC-HDL_, the total cholesterol to high-density lipoprotein cholesterol ratio.

**Table 7:** Multiple regression coefficients for digital measures of Simple Reaction Time task.

Clinical risk factors	ART_all	ART_correct	ART_test	MRT_test	SDRT_test	CE	Percent_correct	SDRT_correct
DBP	2.3 (0.1, 4.5) 0.04	2.2 (0.2, 4.2) 0.03	2.3 (0.1, 4.5) 0.04	2.3 (0.0, 4.7) 0.05		−0.8 (−1.5, −0.0) 0.05		
MI	155.7 (62.0, 249.5) 0.00	206.2 (105.7, 306.7) 0.00	155.8 (62.2, 249.5) 0.00	152.2 (54.7, 249.7) 0.00		−46.6 (−78.9, −14.2) 0.01		
Stroke	149.7 (38.0, 261.3) 0.01	138.8 (39.6, 237.9) 0.01	149.7 (38.1, 261.3) 0.01	134.1 (18.0, 250.3) 0.02		−50.7 (−89.3, −12.2) 0.01		
CHF		−177.8 (−348.2, −7.3) 0.04			83.2 (12.5, 154.0) 0.02			49.2 (1.9, 96.6) 0.04
Total cholesterol					−0.3 (−0.6, −0.1) 0.02			−0.3 (−0.4, −0.1) 0.01
Ratio_TC-HDL_					11.8 (1.4, 22.1) 0.03			10.0 (3.0, 16.9) 0.01
SBP							−0.2 (−0.3, −0.1) 0.00	

The values are presented as coefficient, 95% confidence interval, *p*-value. Blank cells indicate that the corresponding clinical factors were not statistically significant and were therefore removed from the final backward models. Regression coefficients are presented per unit for continuous variables or as the presence of a condition for binary variables. Age and sex were forced as covariates in all the models. DBP, diastolic blood pressure; MI, myocardial infarction; CHF, congestive heart failure; Ratio_TC-HDL_, the total cholesterol to high-density lipoprotein cholesterol ratio; SBP, systolic blood pressure.

## 4 Discussion

In this study, we established the reference values for digital neuropsychological measures for three DANA tasks in a large community-based cohort. These reference values were categorized by three age intervals, separately for men and women. Our findings provide new insights into the digital cognitive performance of individuals in different age groups.

Our results align with previous research in the field of cognitive assessment. Similar to our observations, studies have reported sex-based differences in cognitive function, where women often outperform men in tasks involving verbal memory and attention (23, 24), such as the Code Substitution task (3, 25). A possible explanation for this difference between sex is organization of memory, with is more bilateral in females, but left-lateralized in the male brain (26). It is suggested that women may have higher connectivity between social motivation, attention, and memory subnetworks (27). However, these gender differences tend to diminish or reverse in tasks requiring spatial processing and reaction time (28, 29), such as the Go-No-Go and Simple Reaction Time tasks (30, 31). It is proposed that males perform better in spatial processing or orientation tasks due to a preference for Euclidian strategies, or two-dimensional geometry (28). Other potential contributing factors include environmental context. For instance, males were more inclined to have greater familiarity with 3D computer simulations used to assess spatial processing, possibly owing to their more prevalent exposure to video games (28, 29). Furthermore, our study adds to the existing body of research by providing comprehensive age-specific reference values. These findings are consistent with prior investigations that have highlighted the impact of aging on cognitive performance (32, 33). The observed decline in reaction times with advancing age echoes the well-documented age-related cognitive changes seen in both sexes (34, 35).

Our study also identified clinical risk factors associated with digital neuropsychological measures across three DANA tasks, revealing noteworthy distinctions among them. Particularly, the average response time, a key neuropsychological measure, exhibited distinct clinical correlates within each task. Our findings regarding the associations between average response time and lipid profile (including total cholesterol) in Code Substitution task were in line with prior studies that have explored the relationship between cholesterol levels and cognitive function (36, 37). Notably, higher total cholesterol levels have been linked to cognitive impairment, emphasizing the importance of cardiovascular health in cognitive outcomes. The positive association between average response time and the prevalence of myocardial infarction and stroke in the Simple Reaction Time task underscores the intricate interplay between cardiovascular health and cognitive processing speed (38, 39). These findings aligned with studies emphasizing the impact of cardiovascular risk factors on cognitive function. Moreover, our analysis revealed that different digital cognitive measures were significantly associated with varying numbers of clinical risk factors across the three DANA tasks. These results emphasize the multifaceted nature of cognitive assessment and highlight the need for a comprehensive evaluation of clinical risk factors in understanding cognitive performance. In the Code Substitution task, there was a negative correlation observed between the average response time for all correct trials and levels of LDL and triglycerides. The relationship between LDL, triglycerides, and cognitive performance has yielded mixed outcomes in various studies. While several research findings indicate a link between high LDL cholesterol and a heightened risk of dementia or reduced cognitive function (40–43), other studies have reported contrary effects (44, 45), or found no conclusive correlation (46). In the case of triglycerides, some investigations suggest that elevated triglyceride levels correlate with cognitive decline (47) and diminished cognitive performance (48). However, other study has not established a connection with dementia or cognitive decline (49). These disparate findings suggest that there are various factors at play, including reverse causation and age effects, in the relationship between LDL, triglycerides, and cognitive function, pointing to the need for more comprehensive data in future research to unravel these mechanisms.

This study has several advantages. First, the study employed eight digital measures from each of the three DANA tasks in a large well-characterized cohort, offering a comprehensive digital assessment of participants’ decision-making and reaction times. These measures provided a detailed and multifaceted view of cognitive function, allowing for a more nuanced analysis. An additional cohort was used as an external validation cohort to validate the reference values. Second, a comprehensive list of clinical risk factors has been considered. The study utilized advanced statistical methods, including a backward elimination approach, to investigate clinical correlates. These robust analytical techniques enhance the accuracy and reliability of the study’s results.

We also acknowledge several limitations in this study. The study exclusively involved White participants due to the limited availability of DANA data on other ethnicities from the FHS and BU ADRC. Future efforts should aim to establish reference values for digital cognitive assessments across a more diverse range of ethnic groups. While the study categorizes participants into three age groups (<60, 60–69, and ≥70), this grouping may not capture more nuanced age-related cognitive changes. As more data becomes available, a finer age stratification could provide a more detailed insight of human performance on digital cognitive assessment. Our findings indicate that education level has an influence on individuals’ performance in DANA tasks. To establish reliable reference values stratified by education, further studies with larger sample sizes will be essential. While our research offers valuable insights into cognitive health assessments using the DANA tool, it is crucial to acknowledge that these measurements exclude assessments of episodic memory—a key cognitive domain impacted by AD. This limitation implies that while our findings contribute to understanding specific aspects of cognitive health, they may not comprehensively cover the cognitive landscape affected by AD. Regarding participant recruitment for our study, we extended invitations to all cognitively intact individuals within the FHS cohort. The essential criteria for participation included owning a smartphone, being proficient in English, and having WiFi access. We recognized that the decision to participate could be influenced by the participants’ cognitive and socioeconomic status. Those who chose to partake in the study may have had a relatively better cognitive condition, enabling them to engage with the study requirements. This scenario raises the possibility of selection bias, suggesting that our sample may not entirely represent the broader population of individuals with intact cognition.

In summary, this study provides valuable insights into digital neuropsychological measures, their reference values, and clinical correlates in well-established cohorts. These insights contribute to the growing body of knowledge in the field of digital cognitive assessment and offer valuable guidance for future research and clinical practice.

## Data availability statement

The data analyzed in this study is subject to the following licenses/restrictions: the datasets analyzed for this study could be requested through a formal research application to the Framingham Heart Study. Requests to access these datasets should be directed to https://www.framinghamheartstudy.org/fhs-for-researchers/.

## Ethics statement

The studies involving humans were approved by the Institutional Review Boards of Boston University Medical Center. The studies were conducted in accordance with the local legislation and institutional requirements. The participants provided their written informed consent to participate in this study.

## Author contributions

HD: Conceptualization, Formal analysis, Investigation, Methodology, Project administration, Writing – original draft, Writing – review & editing. MK: Writing – review & editing. ES: Data curation, Software, Writing – review & editing. PS: Writing – review & editing. IA-D: Writing – review & editing. SL: Writing – review & editing. ZP: Writing – review & editing. PH: Writing – review & editing. ZL: Data curation, Writing – review & editing. KG: Writing – review & editing. LH: Writing – review & editing. JM: Writing – review & editing. SR: Writing – review & editing. AI: Writing – review & editing. VK: Writing – review & editing. RA: Writing – review & editing. HL: Conceptualization, Supervision, Writing – review & editing.
