# Corrigendum: Digital neuropsychological measures by defense automated neurocognitive assessment: reference values and clinical correlates

**DOI:** 10.3389/fneur.2024.1433972

**Published:** 2024-05-29

**Authors:** Huitong Ding, Minzae Kim, Edward Searls, Preeti Sunderaraman, Ileana De Anda-Duran, Spencer Low, Zachary Popp, Phillip H. Hwang, Zexu Li, Kriti Goyal, Lindsay Hathaway, Jose Monteverde, Salman Rahman, Akwaugo Igwe, Vijaya B. Kolachalama, Rhoda Au, Honghuang Lin

**Affiliations:** ^1^Department of Anatomy and Neurobiology, Boston University Chobanian & Avedisian School of Medicine, Boston, MA, United States; ^2^The Framingham Heart Study, Boston University Chobanian &Avedisian School of Medicine, Boston, MA, United States; ^3^Department of Neurology, Boston University Chobanian & Avedisian School of Medicine, Boston, MA, United States; ^4^School of Public Health and Tropical Medicine, Tulane University, New Orleans, LA, United States; ^5^Department of Epidemiology, Boston University School of Public Health, Boston, MA, United States; ^6^Department of Medicine, Boston University Chobanian & Avedisian School of Medicine, Boston, MA, United States; ^7^Department of Computer Science, Faculty of Computing & Data Sciences, Boston University, Boston, MA, United States; ^8^Slone Epidemiology Center, Boston University Chobanian & Avedisian School of Medicine, Boston, MA, United States; ^9^Department of Medicine, University of Massachusetts Chan Medical School, Worcester, MA, United States

**Keywords:** cognitive health, defense automated neurocognitive assessment, digital neuropsychological measures, reference values, clinical correlates

In the published article, there was an error in the Funding statement. The following grant was not included: “National Institute on Aging grant number - 2P30AG013846”.

The original text was: The author(s) declare financial support was received for the research, authorship, and/or publication of this article. This work was supported by the National Heart, Lung, and Blood Institute Contract (N01-HC-25195) and by grants from the National Institute on Aging AG-008122, AG-16495, AG-062109, AG-049810, AG-068753, AG054156, P30AG073107, R01AG080670, U01AG068221 and from the National Institute of Neurological Disorders and Stroke, NS017950, and from the Alzheimer's Association (Grant AARG-NTF-20-643020), and the American Heart Association (20SFRN35360180). It was also supported by Defense Advanced Research Projects Agency contract (FA8750-16-C-0299); Pfizer, Inc. The funding agencies had no role in study design, data collection and analysis, decision to publish, or preparation of the manuscript.

The correct Funding statement appears below.

## Funding

The author(s) declare financial support was received for the research, authorship, and/or publication of this article. This work was supported by the National Heart, Lung, and Blood Institute Contract (N01-HC-25195) and by grants from the National Institute on Aging AG-008122, AG-16495, AG-062109, AG-049810, AG-068753, AG054156, 2P30AG013846, P30AG073107, R01AG080670, U01AG068221 and from the National Institute of Neurological Disorders and Stroke, NS017950, and from the Alzheimer's Association (Grant AARG-NTF-20-643020), and the American Heart Association (20SFRN35360180). It was also supported by Defense Advanced Research Projects Agency contract (FA8750-16-C-0299); Pfizer, Inc. The funding agencies had no role in study design, data collection and analysis, decision to publish, or preparation of the manuscript.

The authors apologize for this error and state that this does not change the scientific conclusions of the article in any way. The original article has been updated.

