# Supplementary material for: Digital neuropsychological measures by defense automated neurocognitive assessment: reference values and clinical correlates
Source: Front Neurol. 2024 Feb 15;15:1340710. doi: 10.3389/fneur.2024.1340710 (PMC10902432; doi:10.3389/fneur.2024.1340710)

Supplementary Material

# Supplementary Data

**Supplementary Table 1.** Comparison of reference values for three DANA tasks in the age group below 60 years. Median (50th percentile) values presented.

| **Digital measure** | **Men** | | | **Women** | | |
| --- | --- | --- | --- | --- | --- | --- |
|  | **CS** | **GNG** | **SRT** | **CS** | **GNG** | **SRT** |
| ART_all | 1660 | 796 | 335 | 1661 | 823 | 334 |
| ART_correct | 1674 | 551 | 334 | 1664 | 575 | 336 |
| ART_test | 1660 | 796 | 334 | 1662 | 823 | 334 |
| MRT_test | 1618 | 569 | 325 | 1610 | 603 | 325 |
| SDRT_test | 398 | 432 | 64 | 382 | 428 | 60 |
| CE | 35 | 107 | 180 | 35 | 104 | 178 |
| Percent_correct | 97 | 100 | 100 | 100 | 100 | 100 |
| SDRT_correct | 394 | 89 | 63 | 380 | 95 | 59 |

CS: Code Substitution; GNG: Go-No-Go; SRT: Simple Reaction Time.

**Supplementary Table 2.** Comparison of reference values for three DANA tasks in the age group between 60 and 69 years. Median (50th percentile) values presented.

| **Digital measure** | **Men** | | | **Women** | | |
| --- | --- | --- | --- | --- | --- | --- |
|  | **CS** | **GNG** | **SRT** | **CS** | **GNG** | **SRT** |
| ART_all | 1898 | 842 | 353 | 1884 | 862 | 361 |
| ART_correct | 1893 | 601 | 348 | 1880 | 625 | 359 |
| ART_test | 1898 | 842 | 353 | 1884 | 862 | 360 |
| MRT_test | 1819 | 641 | 336 | 1836 | 666 | 343 |
| SDRT_test | 429 | 414 | 73 | 463 | 410 | 78 |
| CE | 31 | 98 | 170 | 31 | 95 | 166 |
| Percent_correct | 97 | 100 | 100 | 97 | 100 | 100 |
| SDRT_correct | 416 | 109 | 72 | 417 | 108 | 72 |

CS: Code Substitution; GNG: Go-No-Go; SRT: Simple Reaction Time.

**Supplementary Table 3.** Comparison of reference values for three DANA tasks in the age group 70 years or older. Median (50th Percentile) values presented.

| **Digital measure** | **Men** | | | **Women** | | |
| --- | --- | --- | --- | --- | --- | --- |
|  | **CS** | **GNG** | **SRT** | **CS** | **GNG** | **SRT** |
| ART_all | 2325 | 892 | 377 | 2137 | 928 | 389 |
| ART_correct | 2285 | 668 | 372 | 2116 | 722 | 384 |
| ART_test | 2325 | 892 | 377 | 2137 | 928 | 390 |
| MRT_test | 2269 | 716 | 355 | 2071 | 784 | 374 |
| SDRT_test | 522 | 392 | 86 | 470 | 365 | 82 |
| CE | 25 | 89 | 159 | 27 | 81 | 154 |
| Percent_correct | 97 | 100 | 100 | 97 | 100 | 100 |
| SDRT_correct | 470 | 126 | 72 | 442 | 131 | 74 |

CS: Code Substitution; GNG: Go-No-Go; SRT: Simple Reaction Time.

**Supplementary Table 4**. Reference values of digital measures in Code Substitution task in participants with college and higher degree.

| **Digital measure** | **Age group** | **Men** | | | | | **Women** | | | | |
| --- | --- | --- | --- | --- | --- | --- | --- | --- | --- | --- | --- |
|  |  | **2.5%** | **25%** | **50%** | **75%** | **97.5%** | **2.5%** | **25%** | **50%** | **75%** | **97.5%** |
| ART_all | <60 (n=259) | 1148 | 1455 | 1631 | 1843 | 2247 | 1228 | 1505 | 1645 | 1827 | 2284 |
|  | 60–69 (n=226) | 1386 | 1667 | 1895 | 2109 | 2542 | 1410 | 1684 | 1845 | 2131 | 2514 |
|  | >=70 (n=136) | 1576 | 2085 | 2276 | 2508 | 2970 | 1636 | 1904 | 2097 | 2342 | 2734 |
| ART_correct | <60 | 1149 | 1469 | 1636 | 1825 | 2251 | 1228 | 1506 | 1645 | 1826 | 2284 |
|  | 60–69 | 1393 | 1668 | 1893 | 2097 | 2538 | 1411 | 1682 | 1830 | 2100 | 2463 |
|  | >=70 | 1576 | 2088 | 2252 | 2471 | 2843 | 1633 | 1910 | 2093 | 2314 | 2705 |
| ART_test | <60 | 1149 | 1456 | 1632 | 1843 | 2247 | 1229 | 1505 | 1645 | 1827 | 2284 |
|  | 60–69 | 1386 | 1667 | 1895 | 2109 | 2542 | 1410 | 1684 | 1845 | 2131 | 2514 |
|  | >=70 | 1576 | 2085 | 2276 | 2508 | 2970 | 1636 | 1904 | 2097 | 2342 | 2734 |
| MRT_test | <60 | 1121 | 1420 | 1564 | 1782 | 2186 | 1150 | 1442 | 1585 | 1788 | 2260 |
|  | 60–69 | 1319 | 1608 | 1820 | 2059 | 2480 | 1377 | 1625 | 1797 | 2024 | 2371 |
|  | >=70 | 1541 | 1987 | 2209 | 2414 | 2920 | 1597 | 1816 | 2062 | 2264 | 2724 |
| SDRT_test | <60 | 198 | 291 | 392 | 484 | 699 | 230 | 310 | 378 | 454 | 647 |
|  | 60–69 | 248 | 336 | 419 | 512 | 691 | 232 | 334 | 430 | 524 | 716 |
|  | >=70 | 289 | 425 | 516 | 584 | 703 | 292 | 390 | 454 | 556 | 751 |
| CE | <60 | 25 | 31 | 36 | 39 | 50 | 26 | 32 | 36 | 40 | 49 |
|  | 60–69 | 21 | 28 | 31 | 35 | 42 | 23 | 28 | 32 | 35 | 43 |
|  | >=70 | 18 | 23 | 26 | 28 | 38 | 19 | 24 | 28 | 31 | 36 |
| Percent_correct | <60 | 88 | 97 | 97 | 100 | 100 | 89 | 97 | 100 | 100 | 100 |
|  | 60–69 | 88 | 94 | 97 | 100 | 100 | 86 | 94 | 97 | 100 | 100 |
|  | >=70 | 81 | 94 | 97 | 100 | 100 | 81 | 94 | 97 | 100 | 100 |
| SDRT_correct | <60 | 199 | 291 | 380 | 468 | 647 | 230 | 306 | 376 | 434 | 585 |
|  | 60–69 | 250 | 333 | 412 | 480 | 639 | 231 | 331 | 401 | 495 | 651 |
|  | >=70 | 289 | 404 | 470 | 549 | 654 | 264 | 367 | 441 | 508 | 665 |

ART_all: Average response time for all trials (ms); ART_correct: Average response time for all correct test trials (ms); SDRT_correct: Standard deviation of response time for correct test trials (ms); ART_test: Average response time for all test trials (ms); MRT_test: Median response time for all test trials (ms); SDRT_test: Standard deviation of response time for all test trials (ms); CE: Cognitive efficiency value (measure of both speed and accuracy); Percent_correct: Percent of trials with correct responses within allocated time.

**Supplementary Table 5.** Reference values of digital measures in Go-No-Go task in participants with college and higher degree.

| **Digital measure** | **Age group** | **Men** | | | | | **Women** | | | | |
| --- | --- | --- | --- | --- | --- | --- | --- | --- | --- | --- | --- |
|  |  | **2.5%** | **25%** | **50%** | **75%** | **97.5%** | **2.5%** | **25%** | **50%** | **75%** | **97.5%** |
| ART_all | <60 (n=255) | 673 | 745 | 788 | 841 | 995 | 703 | 761 | 820 | 855 | 970 |
|  | 60–69 (n=220) | 713 | 793 | 840 | 893 | 1098 | 732 | 814 | 857 | 919 | 1045 |
|  | >=70 (n=130) | 726 | 855 | 890 | 956 | 1111 | 768 | 858 | 928 | 971 | 1104 |
| ART_correct | <60 | 404 | 479 | 546 | 597 | 799 | 436 | 504 | 568 | 618 | 787 |
|  | 60–69 | 462 | 536 | 597 | 666 | 838 | 492 | 565 | 619 | 693 | 863 |
|  | >=70 | 494 | 613 | 663 | 764 | 963 | 502 | 617 | 720 | 776 | 933 |
| ART_test | <60 | 673 | 745 | 788 | 842 | 995 | 703 | 762 | 820 | 855 | 970 |
|  | 60–69 | 714 | 793 | 840 | 893 | 1098 | 732 | 814 | 857 | 919 | 1045 |
|  | >=70 | 725 | 855 | 890 | 956 | 1111 | 768 | 858 | 928 | 971 | 1103 |
| MRT_test | <60 | 424 | 496 | 568 | 636 | 848 | 452 | 522 | 600 | 666 | 802 |
|  | 60–69 | 486 | 554 | 636 | 714 | 1032 | 501 | 599 | 665 | 752 | 946 |
|  | >=70 | 523 | 640 | 716 | 810 | 1036 | 534 | 648 | 774 | 816 | 1025 |
| SDRT_test | <60 | 322 | 412 | 442 | 464 | 500 | 347 | 406 | 430 | 456 | 483 |
|  | 60–69 | 301 | 391 | 418 | 438 | 479 | 298 | 372 | 411 | 432 | 457 |
|  | >=70 | 269 | 352 | 396 | 416 | 467 | 275 | 344 | 369 | 413 | 473 |
| CE | <60 | 74 | 99 | 109 | 124 | 143 | 75 | 96 | 104 | 118 | 136 |
|  | 60–69 | 70 | 89 | 100 | 109 | 124 | 67 | 86 | 95 | 105 | 121 |
|  | >=70 | 62 | 79 | 90 | 96 | 119 | 61 | 77 | 82 | 97 | 119 |
| Percent_correct | <60 | 90 | 100 | 100 | 100 | 100 | 96 | 100 | 100 | 100 | 100 |
|  | 60–69 | 92 | 97 | 100 | 100 | 100 | 93 | 100 | 100 | 100 | 100 |
|  | >=70 | 90 | 97 | 100 | 100 | 100 | 93 | 100 | 100 | 100 | 100 |
| SDRT_correct | <60 | 47 | 70 | 86 | 114 | 210 | 53 | 77 | 94 | 134 | 204 |
|  | 60–69 | 61 | 86 | 106 | 141 | 197 | 56 | 85 | 105 | 134 | 226 |
|  | >=70 | 72 | 95 | 128 | 156 | 212 | 69 | 105 | 130 | 148 | 218 |

ART_all: Average response time for all trials (ms); ART_correct: Average response time for all correct test trials (ms); SDRT_correct: Standard deviation of response time for correct test trials (ms); ART_test: Average response time for all test trials (ms); MRT_test: Median response time for all test trials (ms); SDRT_test: Standard deviation of response time for all test trials (ms); CE: Cognitive efficiency value (measure of both speed and accuracy); Percent_correct: Percent of trials with correct responses within allocated time.

**Supplementary Table 6.** Reference values of digital measures in Simple Reaction Time task in participants with college and higher degree.

| **Digital measure** | **Age group** | **Men** | | | | | **Women** | | | | |
| --- | --- | --- | --- | --- | --- | --- | --- | --- | --- | --- | --- |
|  |  | **2.5%** | **25%** | **50%** | **75%** | **97.5%** | **2.5%** | **25%** | **50%** | **75%** | **97.5%** |
| ART_all | <60 (n=113) | 257 | 291 | 330 | 369 | 480 | 269 | 314 | 340 | 372 | 497 |
|  | 60–69 (n=119) | 275 | 313 | 351 | 380 | 481 | 266 | 329 | 352 | 389 | 524 |
|  | >=70 (n=67) | 260 | 327 | 358 | 411 | 557 | 302 | 330 | 367 | 415 | 636 |
| ART_correct | <60 | 257 | 292 | 330 | 364 | 476 | 269 | 314 | 341 | 375 | 491 |
|  | 60–69 | 275 | 314 | 346 | 380 | 459 | 266 | 328 | 352 | 389 | 505 |
|  | >=70 | 265 | 327 | 346 | 402 | 548 | 302 | 330 | 367 | 414 | 533 |
| ART_test | <60 | 257 | 291 | 330 | 369 | 479 | 269 | 314 | 340 | 372 | 496 |
|  | 60–69 | 275 | 313 | 351 | 380 | 481 | 266 | 329 | 352 | 389 | 524 |
|  | >=70 | 260 | 327 | 358 | 410 | 557 | 302 | 330 | 367 | 414 | 637 |
| MRT_test | <60 | 248 | 276 | 312 | 355 | 453 | 256 | 304 | 330 | 363 | 485 |
|  | 60–69 | 262 | 295 | 334 | 360 | 476 | 252 | 309 | 338 | 377 | 496 |
|  | >=70 | 250 | 310 | 339 | 388 | 537 | 278 | 312 | 348 | 409 | 629 |
| SDRT_test | <60 | 29 | 51 | 64 | 83 | 199 | 31 | 53 | 62 | 89 | 154 |
|  | 60–69 | 38 | 53 | 66 | 90 | 152 | 35 | 53 | 70 | 86 | 149 |
|  | >=70 | 41 | 68 | 84 | 104 | 135 | 43 | 58 | 70 | 91 | 162 |
| CE | <60 | 118 | 162 | 182 | 204 | 234 | 121 | 156 | 176 | 191 | 223 |
|  | 60–69 | 125 | 158 | 170 | 191 | 215 | 113 | 154 | 170 | 183 | 223 |
|  | >=70 | 106 | 146 | 169 | 184 | 221 | 87 | 145 | 164 | 181 | 198 |
| Percent_correct | <60 | 88 | 100 | 100 | 100 | 100 | 90 | 100 | 100 | 100 | 100 |
|  | 60–69 | 90 | 100 | 100 | 100 | 100 | 95 | 100 | 100 | 100 | 100 |
|  | >=70 | 95 | 98 | 100 | 100 | 100 | 82 | 100 | 100 | 100 | 100 |
| SDRT_correct | <60 | 29 | 51 | 63 | 83 | 132 | 31 | 53 | 62 | 87 | 132 |
|  | 60–69 | 38 | 53 | 64 | 82 | 125 | 35 | 53 | 65 | 86 | 112 |
|  | >=70 | 38 | 58 | 72 | 87 | 122 | 43 | 53 | 68 | 85 | 116 |

ART_all: Average response time for all trials (ms); ART_correct: Average response time for all correct test trials (ms); SDRT_correct: Standard deviation of response time for correct test trials (ms); ART_test: Average response time for all test trials (ms); MRT_test: Median response time for all test trials (ms); SDRT_test: Standard deviation of response time for all test trials (ms); CE: Cognitive efficiency value (measure of both speed and accuracy); Percent_correct: Percent of trials with correct responses within allocated time.

**Supplementary Table 7**. Reference values of digital measures in Code Substitution task in participants with no college degree.

| **Digital measure** | **Age group** | **Men** | | | | | **Women** | | | | |
| --- | --- | --- | --- | --- | --- | --- | --- | --- | --- | --- | --- |
|  |  | **2.5%** | **25%** | **50%** | **75%** | **97.5%** | **2.5%** | **25%** | **50%** | **75%** | **97.5%** |
| ART_all | <60 (n=79) | 1388 | 1616 | 1821 | 2038 | 2531 | 1036 | 1629 | 1763 | 2001 | 2394 |
|  | 60–69 (n=152) | 1368 | 1703 | 1919 | 2170 | 2855 | 1402 | 1802 | 1968 | 2186 | 2625 |
|  | >=70 (n=80) | 1702 | 2219 | 2515 | 2652 | 3021 | 1729 | 1934 | 2223 | 2441 | 2861 |
| ART_correct | <60 | 1388 | 1617 | 1816 | 2004 | 2529 | 1070 | 1626 | 1746 | 1996 | 2313 |
|  | 60–69 | 1377 | 1704 | 1870 | 2157 | 2725 | 1402 | 1795 | 1938 | 2175 | 2562 |
|  | >=70 | 1707 | 2168 | 2446 | 2575 | 2837 | 1721 | 1917 | 2222 | 2424 | 2722 |
| ART_test | <60 | 1388 | 1617 | 1822 | 2038 | 2531 | 1036 | 1628 | 1763 | 2001 | 2394 |
|  | 60–69 | 1368 | 1702 | 1919 | 2170 | 2855 | 1402 | 1802 | 1968 | 2186 | 2625 |
|  | >=70 | 1702 | 2219 | 2514 | 2652 | 3021 | 1730 | 1934 | 2223 | 2442 | 2861 |
| MRT_test | <60 | 1297 | 1611 | 1772 | 1968 | 2559 | 1009 | 1559 | 1701 | 1920 | 2200 |
|  | 60–69 | 1269 | 1609 | 1788 | 2083 | 2718 | 1315 | 1693 | 1896 | 2082 | 2609 |
|  | >=70 | 1640 | 2032 | 2460 | 2627 | 3336 | 1639 | 1858 | 2187 | 2362 | 2796 |
| SDRT_test | <60 | 174 | 356 | 418 | 510 | 625 | 165 | 320 | 403 | 510 | 687 |
|  | 60–69 | 266 | 388 | 476 | 537 | 660 | 246 | 409 | 494 | 564 | 691 |
|  | >=70 | 267 | 421 | 552 | 636 | 791 | 232 | 392 | 498 | 581 | 772 |
| CE | <60 | 21 | 27 | 33 | 36 | 44 | 24 | 30 | 33 | 36 | 50 |
|  | 60–69 | 18 | 27 | 30 | 35 | 42 | 18 | 26 | 30 | 33 | 43 |
|  | >=70 | 16 | 21 | 24 | 27 | 34 | 17 | 23 | 26 | 30 | 34 |
| Percent_correct | <60 | 79 | 94 | 97 | 100 | 100 | 89 | 97 | 100 | 100 | 100 |
|  | 60–69 | 83 | 94 | 97 | 100 | 100 | 82 | 94 | 97 | 100 | 100 |
|  | >=70 | 68 | 92 | 97 | 99 | 100 | 76 | 92 | 97 | 100 | 100 |
| SDRT_correct | <60 | 174 | 325 | 403 | 458 | 585 | 165 | 320 | 395 | 480 | 552 |
|  | 60–69 | 264 | 363 | 422 | 499 | 609 | 246 | 354 | 451 | 524 | 642 |
|  | >=70 | 262 | 399 | 472 | 569 | 605 | 231 | 379 | 446 | 522 | 640 |

ART_all: Average response time for all trials (ms); ART_correct: Average response time for all correct test trials (ms); SDRT_correct: Standard deviation of response time for correct test trials (ms); ART_test: Average response time for all test trials (ms); MRT_test: Median response time for all test trials (ms); SDRT_test: Standard deviation of response time for all test trials (ms); CE: Cognitive efficiency value (measure of both speed and accuracy); Percent_correct: Percent of trials with correct responses within allocated time.

**Supplementary Table 8.** Reference values of digital measures in Go-No-Go task in participants with no college degree.

| **Digital measure** | **Age group** | **Men** | | | | | **Women** | | | | |
| --- | --- | --- | --- | --- | --- | --- | --- | --- | --- | --- | --- |
|  |  | **2.5%** | **25%** | **50%** | **75%** | **97.5%** | **2.5%** | **25%** | **50%** | **75%** | **97.5%** |
| ART_all | <60 (n=78) | 701 | 748 | 812 | 891 | 961 | 701 | 781 | 845 | 882 | 1079 |
|  | 60–69 (n=145) | 726 | 784 | 854 | 920 | 1056 | 688 | 817 | 870 | 933 | 1043 |
|  | >=70 (n=78) | 797 | 840 | 904 | 988 | 1182 | 736 | 862 | 944 | 1034 | 1228 |
| ART_correct | <60 | 425 | 476 | 562 | 654 | 765 | 451 | 520 | 607 | 662 | 898 |
|  | 60–69 | 464 | 552 | 614 | 711 | 833 | 474 | 574 | 642 | 732 | 826 |
|  | >=70 | 542 | 612 | 706 | 802 | 989 | 533 | 630 | 750 | 857 | 1000 |
| ART_test | <60 | 701 | 748 | 812 | 892 | 961 | 701 | 781 | 844 | 882 | 1078 |
|  | 60–69 | 726 | 785 | 854 | 920 | 1056 | 688 | 817 | 870 | 933 | 1042 |
|  | >=70 | 797 | 840 | 904 | 988 | 1181 | 735 | 862 | 944 | 1034 | 1228 |
| MRT_test | <60 | 442 | 504 | 580 | 730 | 812 | 472 | 541 | 638 | 700 | 993 |
|  | 60–69 | 483 | 566 | 653 | 748 | 935 | 484 | 596 | 667 | 763 | 926 |
|  | >=70 | 557 | 644 | 749 | 853 | 1229 | 554 | 652 | 814 | 890 | 1205 |
| SDRT_test | <60 | 353 | 401 | 429 | 467 | 484 | 314 | 390 | 412 | 444 | 474 |
|  | 60–69 | 322 | 368 | 412 | 443 | 477 | 320 | 363 | 405 | 429 | 459 |
|  | >=70 | 274 | 332 | 372 | 416 | 449 | 212 | 310 | 360 | 402 | 447 |
| CE | <60 | 75 | 90 | 104 | 125 | 140 | 65 | 88 | 99 | 116 | 133 |
|  | 60–69 | 67 | 83 | 96 | 107 | 128 | 67 | 80 | 93 | 104 | 122 |
|  | >=70 | 54 | 75 | 82 | 95 | 111 | 52 | 68 | 78 | 90 | 112 |
| Percent_correct | <60 | 90 | 99 | 100 | 100 | 100 | 97 | 100 | 100 | 100 | 100 |
|  | 60–69 | 91 | 97 | 100 | 100 | 100 | 68 | 100 | 100 | 100 | 100 |
|  | >=70 | 84 | 97 | 100 | 100 | 100 | 79 | 97 | 100 | 100 | 100 |
| SDRT_correct | <60 | 46 | 71 | 96 | 122 | 183 | 58 | 80 | 97 | 132 | 196 |
|  | 60–69 | 67 | 86 | 114 | 160 | 221 | 54 | 86 | 114 | 142 | 228 |
|  | >=70 | 65 | 96 | 119 | 156 | 211 | 75 | 105 | 132 | 153 | 238 |

ART_all: Average response time for all trials (ms); ART_correct: Average response time for all correct test trials (ms); SDRT_correct: Standard deviation of response time for correct test trials (ms); ART_test: Average response time for all test trials (ms); MRT_test: Median response time for all test trials (ms); SDRT_test: Standard deviation of response time for all test trials (ms); CE: Cognitive efficiency value (measure of both speed and accuracy); Percent_correct: Percent of trials with correct responses within allocated time.

**Supplementary Table 9.** Reference values of digital measures in Simple Reaction Time task in participants with no college degree.

| **Digital measure** | **Age group** | **Men** | | | | | **Women** | | | | |
| --- | --- | --- | --- | --- | --- | --- | --- | --- | --- | --- | --- |
|  |  | **2.5%** | **25%** | **50%** | **75%** | **97.5%** | **2.5%** | **25%** | **50%** | **75%** | **97.5%** |
| ART_all | <60 (n=36) | 271 | 327 | 343 | 446 | 509 | 255 | 298 | 323 | 368 | 437 |
|  | 60–69 (n=79) | 302 | 332 | 355 | 400 | 505 | 257 | 333 | 377 | 447 | 582 |
|  | >=70 (n=38) | 309 | 352 | 390 | 450 | 597 | 281 | 345 | 420 | 503 | 652 |
| ART_correct | <60 | 271 | 327 | 342 | 443 | 499 | 255 | 298 | 316 | 368 | 438 |
|  | 60–69 | 306 | 331 | 350 | 398 | 498 | 258 | 328 | 375 | 440 | 557 |
|  | >=70 | 308 | 352 | 379 | 432 | 590 | 287 | 341 | 420 | 500 | 644 |
| ART_test | <60 | 271 | 327 | 342 | 446 | 510 | 255 | 298 | 323 | 368 | 438 |
|  | 60–69 | 302 | 331 | 355 | 400 | 505 | 258 | 332 | 377 | 447 | 582 |
|  | >=70 | 308 | 352 | 390 | 450 | 597 | 281 | 344 | 420 | 503 | 652 |
| MRT_test | <60 | 263 | 302 | 337 | 428 | 484 | 246 | 290 | 307 | 346 | 428 |
|  | 60–69 | 285 | 313 | 340 | 387 | 493 | 252 | 315 | 359 | 415 | 536 |
|  | >=70 | 299 | 332 | 372 | 429 | 590 | 270 | 332 | 397 | 484 | 639 |
| SDRT_test | <60 | 44 | 50 | 65 | 98 | 142 | 27 | 44 | 56 | 71 | 109 |
|  | 60–69 | 48 | 69 | 88 | 113 | 158 | 35 | 63 | 85 | 120 | 188 |
|  | >=70 | 46 | 77 | 93 | 105 | 145 | 48 | 72 | 90 | 121 | 158 |
| CE | <60 | 117 | 134 | 175 | 184 | 222 | 137 | 164 | 188 | 202 | 236 |
|  | 60–69 | 119 | 151 | 164 | 180 | 192 | 100 | 135 | 159 | 179 | 233 |
|  | >=70 | 101 | 134 | 156 | 170 | 194 | 90 | 120 | 142 | 174 | 204 |
| Percent_correct | <60 | 98 | 100 | 100 | 100 | 100 | 99 | 100 | 100 | 100 | 100 |
|  | 60–69 | 90 | 100 | 100 | 100 | 100 | 90 | 98 | 100 | 100 | 100 |
|  | >=70 | 95 | 98 | 100 | 100 | 100 | 94 | 98 | 100 | 100 | 100 |
| SDRT_correct | <60 | 44 | 50 | 60 | 98 | 128 | 27 | 44 | 56 | 67 | 106 |
|  | 60–69 | 48 | 66 | 85 | 97 | 122 | 35 | 57 | 81 | 96 | 121 |
|  | >=70 | 39 | 68 | 80 | 94 | 122 | 48 | 70 | 83 | 93 | 137 |

ART_all: Average response time for all trials (ms); ART_correct: Average response time for all correct test trials (ms); SDRT_correct: Standard deviation of response time for correct test trials (ms); ART_test: Average response time for all test trials (ms); MRT_test: Median response time for all test trials (ms); SDRT_test: Standard deviation of response time for all test trials (ms); CE: Cognitive efficiency value (measure of both speed and accuracy); Percent_correct: Percent of trials with correct responses within allocated time.

**Supplementary Table 10.** Skewness for each of the partitions for three DANA tasks.

| **Digital measure** | **Age group** | **Code Substitution** | | **Go-No-Go** | | **Simple Reaction Time** | |
| --- | --- | --- | --- | --- | --- | --- | --- |
|  |  | **Men** | **Women** | **Men** | **Women** | **Men** | **Women** |
| ART_all | <60 | 0.40 | 0.30 | 1.77 | 0.77 | 1.00 | 1.34 |
|  | 60–69 | 0.60 | 0.50 | 1.52 | 1.06 | 0.96 | 1.13 |
|  | >=70 | 0.45 | 0.23 | 1.09 | 0.54 | 1.27 | 1.11 |
| ART_correct | <60 | 0.39 | 0.24 | 0.78 | 0.95 | 0.94 | 1.28 |
|  | 60–69 | 0.43 | 0.36 | 0.66 | 0.54 | 0.91 | 1.14 |
|  | >=70 | -0.32 | 0.16 | 0.74 | 0.36 | 1.40 | 1.02 |
| ART_test | <60 | 0.40 | 0.30 | 1.77 | 0.77 | 1.00 | 1.34 |
|  | 60–69 | 0.60 | 0.50 | 1.52 | 1.06 | 0.96 | 1.13 |
|  | >=70 | 0.45 | 0.23 | 1.09 | 0.54 | 1.27 | 1.10 |
| MRT_test | <60 | 0.47 | 0.21 | 2.65 | 1.02 | 0.92 | 1.50 |
|  | 60–69 | 0.69 | 0.57 | 2.68 | 2.13 | 1.11 | 1.16 |
|  | >=70 | 1.07 | 0.32 | 2.00 | 1.04 | 1.46 | 1.20 |
| SDRT_test | <60 | 0.33 | 0.77 | -0.77 | -0.80 | 1.74 | 1.36 |
|  | 60–69 | 0.56 | 0.67 | -0.64 | -0.75 | 0.94 | 1.09 |
|  | >=70 | 0.10 | 0.72 | -0.58 | -0.40 | 0.15 | 1.33 |
| CE | <60 | 0.42 | 0.54 | -0.37 | 0.00 | -0.27 | -0.05 |
|  | 60–69 | -0.15 | -0.09 | -0.72 | -0.30 | -0.26 | -0.03 |
|  | >=70 | -0.28 | -0.10 | -0.59 | 0.53 | -0.14 | -0.42 |
| Percent_correct | <60 | -5.10 | -5.60 | -8.35 | -3.65 | -5.24 | -4.59 |
|  | 60–69 | -5.38 | -4.69 | -7.66 | -5.41 | -4.03 | -2.44 |
|  | >=70 | -5.46 | -3.13 | -7.12 | -5.72 | -1.93 | -6.11 |
| SDRT_correct | <60 | 0.20 | 0.37 | 1.09 | 0.81 | 1.24 | 0.92 |
|  | 60–69 | 0.68 | 0.55 | 0.97 | 0.92 | 0.69 | 0.81 |
|  | >=70 | 0.15 | 0.24 | 0.47 | 0.82 | 0.28 | 0.59 |

**Supplementary Figure 1.** The AIC values across iterations in the stepwise elimination procedure for Code Substitution task.

***
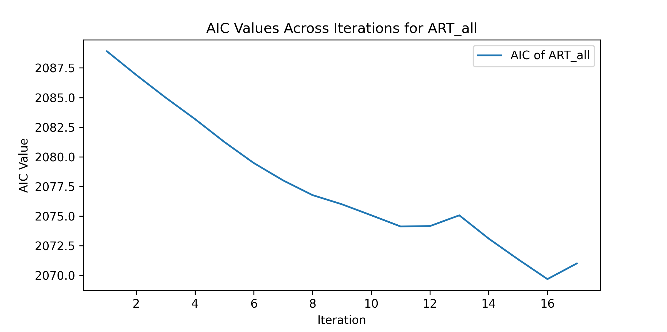

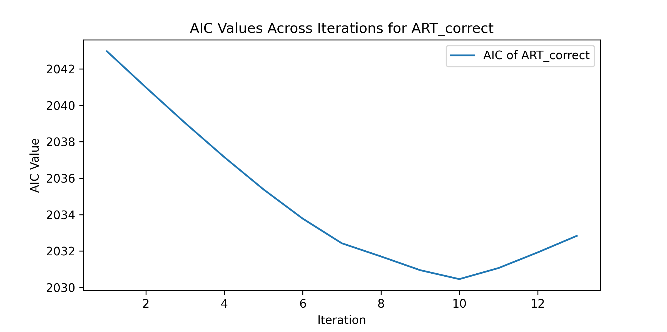

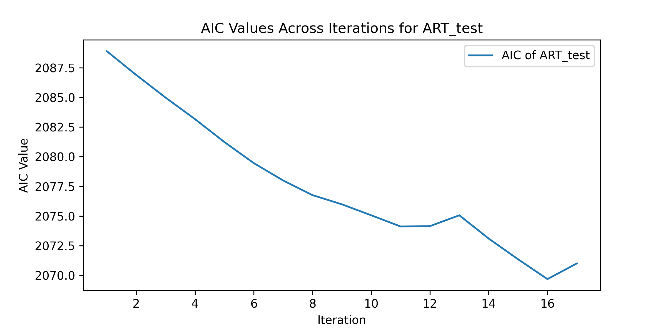
***

***
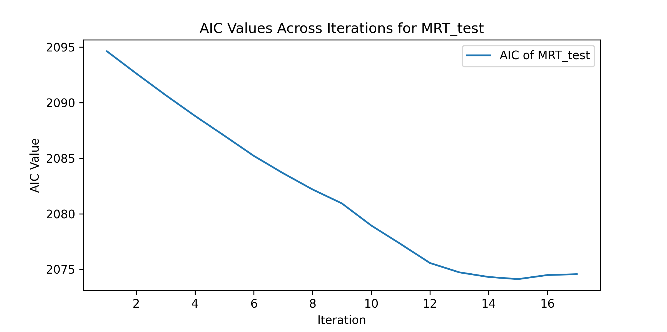

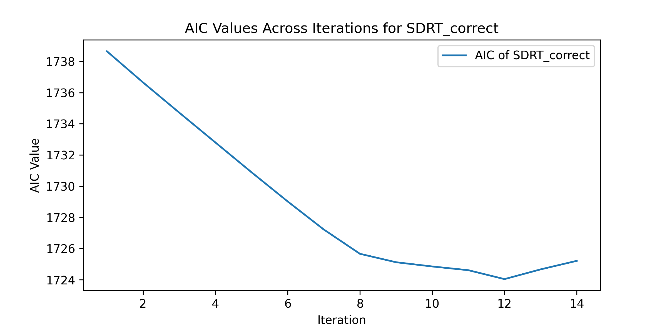

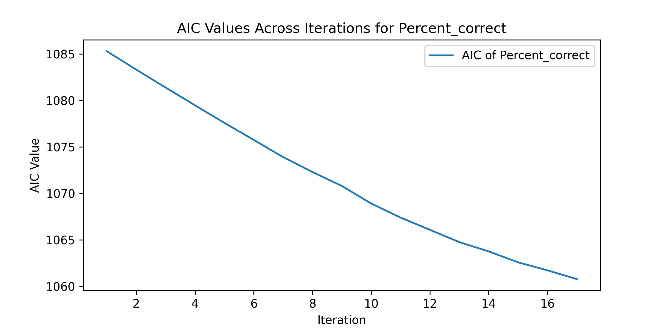
***

***
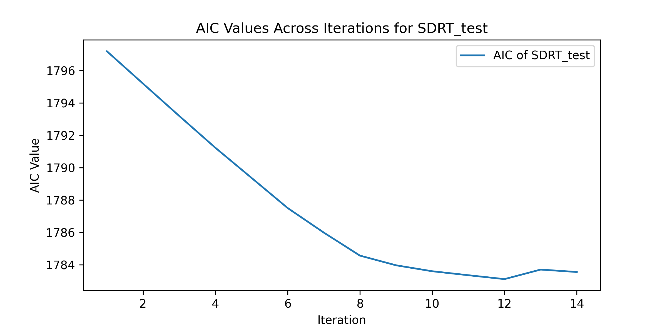
***

**Supplementary Figure 2.** The AIC values across iterations in the stepwise elimination procedure for Go-No-Go task.


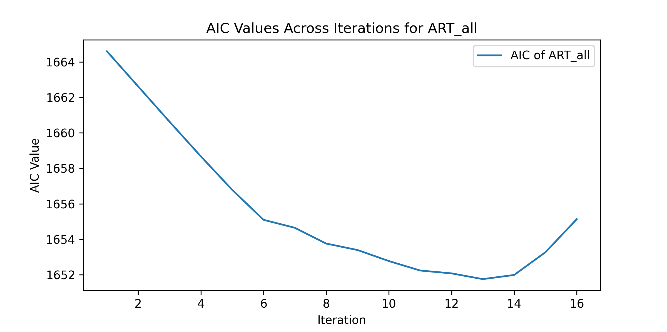

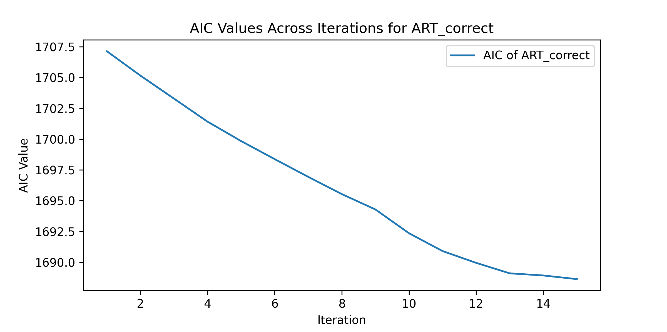

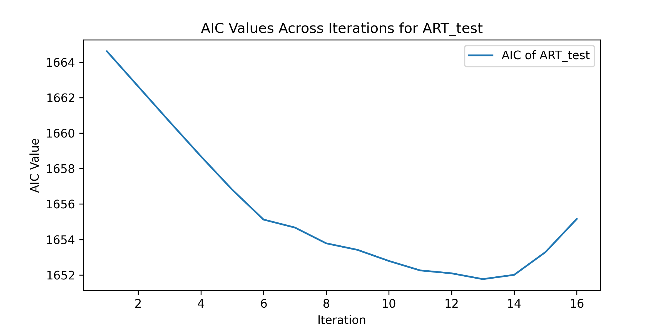


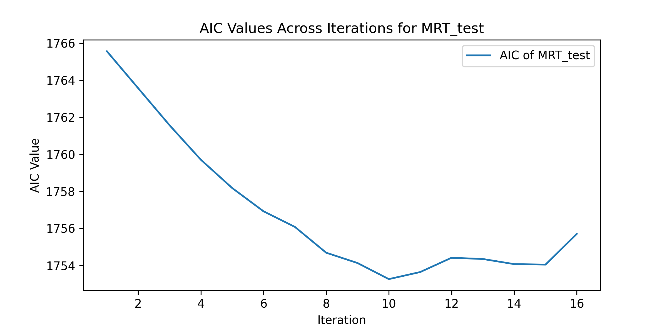

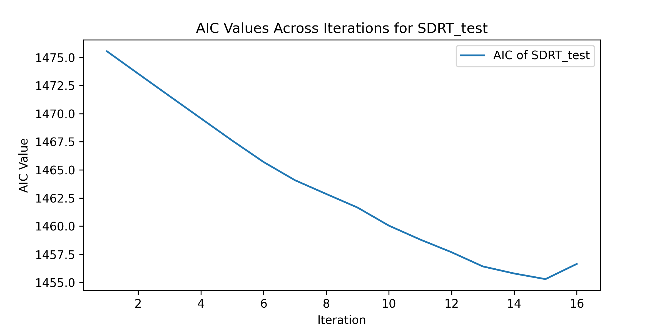

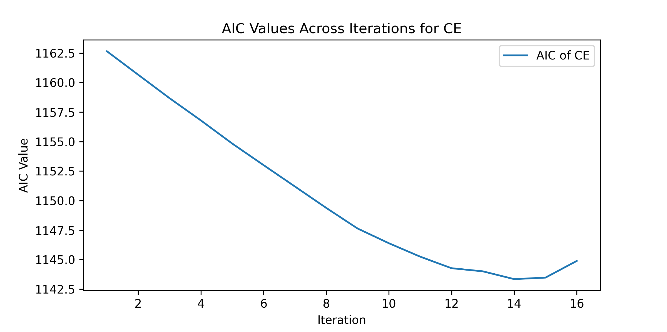


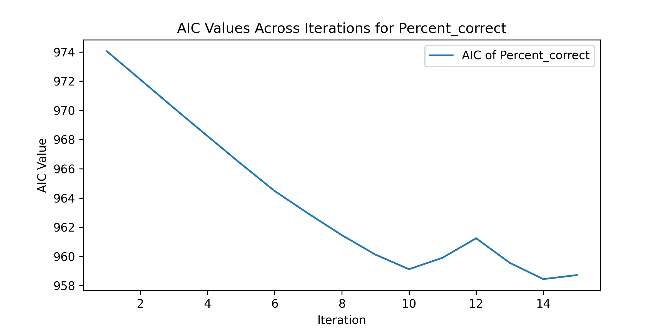


**Supplementary Figure 3.** The AIC values across iterations in the stepwise elimination procedure for Simple Reaction Time task.


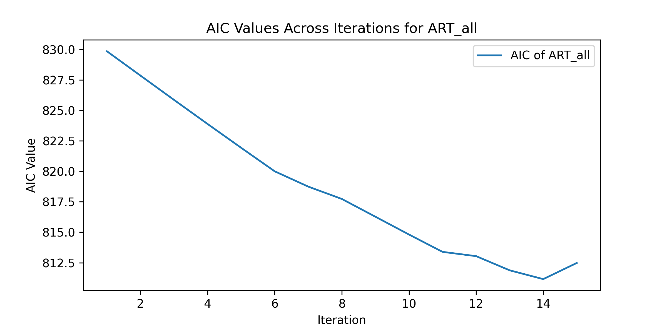

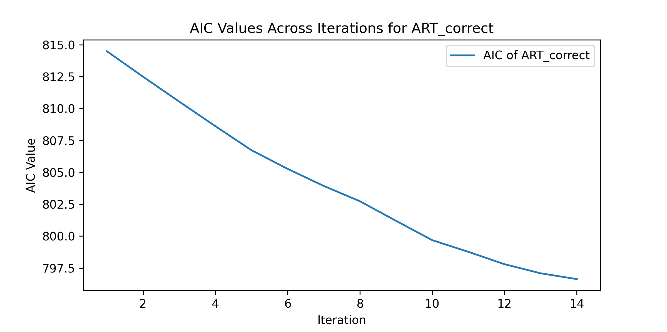

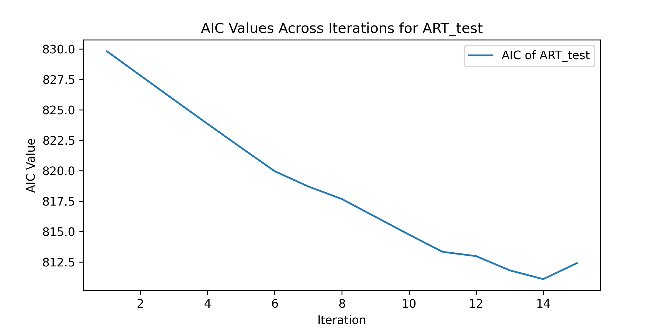


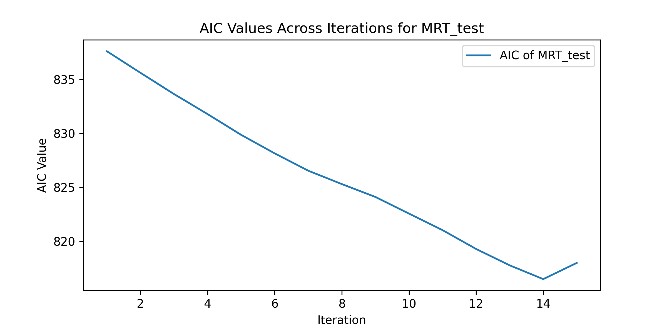

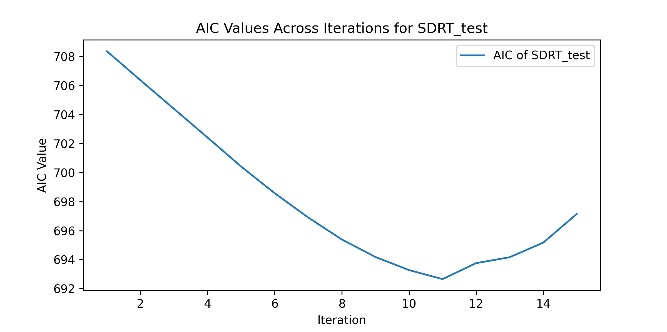

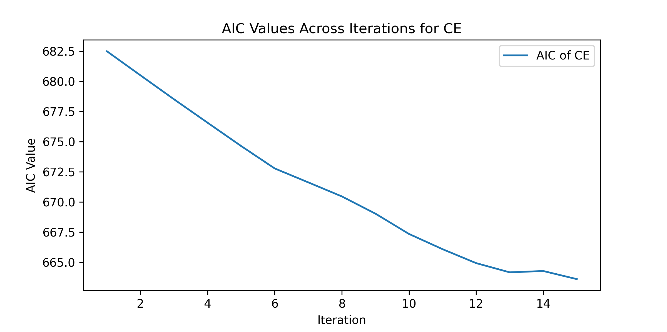


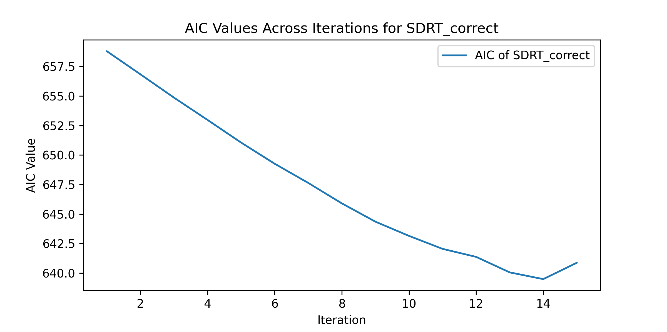

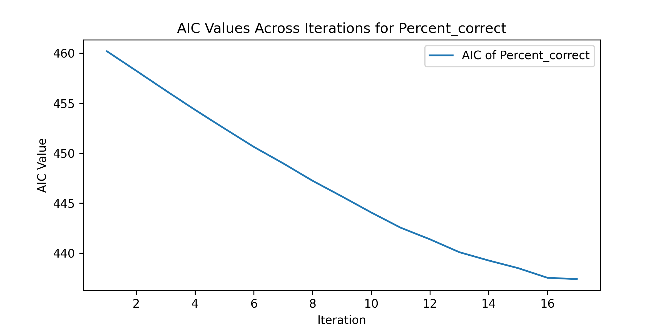

Supplement: Supplementary file 1 [file Table_1.DOCX]
